# Diet, Exercise and Gestational Diabetes Mellitus

**DOI:** 10.3390/nu15102251

**Published:** 2023-05-10

**Authors:** Trine Moholdt

**Affiliations:** 1 Department of Circulation and Medical Imaging, Norwegian University of Science and Technology, 7030 Trondheim, Norway; trine.moholdt@ntnu.no; 2 Women’s Clinic, St. Olavs Hospital, 7030 Trondheim, Norway

Gestational diabetes mellitus (GDM) is defined as hyperglycaemia with blood glucose values above normal, but below those diagnostic of diabetes, and is the most common metabolic disease in pregnancy [1]. The incidence of GDM depends on the population and diagnostic criteria, and current estimates indicate that around 15% of pregnant people globally are affected [2]. The prevalence of GDM is strongly associated with maternal body mass index (BMI), with the risk being four times higher in women with obesity (BMI ≥ 30 kg/m^2^) compared with women who have a healthy BMI (18.5–24.9 kg/m^2^). GDM is a growing health concern due to its close association with increasing BMI and with adverse pregnancy outcomes for the mother and offspring [3,4,5]. Lifestyle modification, including both diet and physical activity, is the first-line option for both the prevention and treatment of GDM [6]. This Special Issue of *Nutrients* includes one umbrella review on the prevention of GDM by healthy diet and/or physical activity during pregnancy, one randomised clinical trial on the prevention of GDM, and three observational studies.

Ellerbrock and colleagues investigated how insulin resistance and beta cell function are related in women with GDM [7]. In a cohort of 2112 second-trimester participants, they determined the relationship between insulin resistance (HOMA-IR), beta cell function (HOMA-β), and the prevalence of abnormal glucose handling. Their results showed that both insulin resistance and beta cell dysfunction were associated with an increased risk of GDM. However, the contribution of low beta cell function was more pronounced for hyperglycaemia in participants without obesity. Based on this finding, the authors argue that it is mainly insulin resistance that underlies the need for glucose lowering medication, especially for people with obesity. These results may have clinical implications for the choice of medication for people with GDM. In their view, based on the dominant underlying mechanism of GDM (insulin resistance), metformin instead of insulin would be the most effective medication option. However, for the *prevention* of GDM, another article included in this Special Issue showed no benefit of adding metformin to a dietary intervention among Mexican pregnant people with high risk of GDM [8]. In a randomised clinical trial, Perichart-Perera and colleagues assigned participants to either intensive medical nutrition therapy alone or with added metformin. In the diet + metformin group, 11 of 45 participants (24.4%) developed GDM, whereas the incidence was 15.5% (7 of 45) in the isolated nutrition therapy group.

Even if dietary modification is a cornerstone in GDM prevention, there is no clear consensus of which dietary pattern is the best. In an umbrella review of systematic reviews and meta-analyses of randomized clinical trials, Kouiti et al. [9] evaluated the effects of diet and/or physical activity interventions during pregnancy on preventing GDM. They found that the protective effects of an isolated dietary intervention were very variable between the systematic reviews that they included in their umbrella review. The Mediterranean diet seems to have the most evidence for GDM prevention, however, many of the included reviews had low or critically low quality as rated using the AMSTAR 2 tool [10]. In addition, for physical activity interventions, the quality of most systematic reviews was rated as low or critically low. However, those reviews that had a moderate or high quality reported that physical activity interventions reduced the risk of GDM by around 40%. For combined interventions, in which both diet and physical activity components were included, the evidence of effect is less clear.

Garnæs and colleagues investigated adherence to nutritional recommendations in early pregnancy and examined associations between early pregnancy dietary intake and late pregnancy glycaemia in pregnant people with obesity [11]. They included 120 participants with pre-pregnancy BMI ≥ 30 kg/m^2^ from two randomised controlled trials of exercise training in pregnancy. As determined by a food frequency questionnaire, 90% of the participants reported early pregnancy dietary intake in accordance with the 2012 Nordic Nutrition Recommendations (NNR) [12]. However, average intakes of vitamin D, iron, and folate were below recommended levels, with only 21% reporting to consume the recommended amount of iron. The authors found no clear association between single dietary variables and GDM diagnosis in late pregnancy, but that early pregnancy intakes of dairy products and protein were associated with lower fasting glucose.

One of the adverse outcomes of GDM is the increased risk of macrosomic (>4000 g) new-borns. In studies with no insulin use, participants with GDM had an odds ratio of 1.70 for the delivery of a macrosomic infant, whereas this number was 1.48 in studies that did not report the use of insulin [5]. Large for gestational age (LGA) is another term applied to excessive foetal growth and generally indicates a birth weight equal to or more than the 90th percentile for a given gestational age. In a prospective study of more than 30,000 pregnancies, Song et al. [13] estimated the proportions of macrosomia and LGA mediated by GDM. Their study showed that GDM might act as a potential mediator for the higher prevalence of macrosomia and LGA in pregnant people who are overweight or obese. The proportion mediated, which answers the question of how much of the total association was explained by GDM, was the highest among those who were overweight, reaching 47% for macrosomia and 30% for LGA births. The mechanisms through which maternal overweight/obesity and hyperglycaemia, isolated or combined, influence the intrauterine environment and foetal growth remain unknown. According to the Pedersen hypothesis [14], the macrosomia of infants born to pregnant people with GDM reflects both foetal hyperglycaemia and subsequent hyperinsulinaemia caused by maternal hyperglycaemia, implying that the growth-promoting actions of both glucose and insulin are responsible for overgrowth.

The studies featured in this Special Issue, together with the wider scientific literature at large, are critical in furthering the evidence base for the clinical management of GDM, the pathophysiological mechanisms underlying this metabolic disorder, as well as its associated consequences regarding foetal growth. However, several gaps in our knowledge still remain to be filled. There is currently limited knowledge on which lifestyle intervention is optimal for preventing the rising prevalence of GDM. Moreover, we have limited evidence for the effect of diet or exercise interventions as stand-alone therapies for GDM. One fruitful topic for further studies should be that of preconception interventions, as current lifestyle interventions have been met with limited success due to implementation too late in pregnancy and poor adherence [15].
